# A Smartphone-Based Psychological Intervention for Nonsuicidal Self-Injury (Kalmer App): Protocol for a Multicenter Double-Blind Randomized Controlled Trial

**DOI:** 10.2196/86413

**Published:** 2026-01-27

**Authors:** Anna Julià, Irene Jaén, Mónica Conesa Giménez, Azucena García-Palacios, Juan C Pascual, Anna Sintes Estévez, Anaís Lara, Iria Méndez, Soledad Romero, Joaquim Puntí, Joaquim Soler, Jordi Solé-Casals, Marina López-Solà, Daniel Vega

**Affiliations:** 1 Fundació Sanitària d’Igualada Igualada Spain; 2 Department of Psychiatry and Forensic Medicine School of Medicine Universitat Autònoma de Barcelona Barcelona Spain; 3 Department of Psychiatry Hospital Universitari d’Igualada Consorci Sanitari de l'Anoia Igualada Spain; 4 Department of Developmental, Educational, Social and Methodology Psychology Universitat Jaume I Castellón de la Plana Spain; 5 Department of Basic and Clinical Psychology and Psychobiology Universitat Jaume I Castellón de la Plana Spain; 6 CIBER de Physiopathology of Obesity and Nutrition (CIBEROBN) Instituto de Salud Carlos III Madrid Spain; 7 Mental Health and Psychiatry Department Vic Hospital Consortium Vic Spain; 8 Centro de Investigación Biomédica en Red de Salud Mental Instituto de Salud Carlos III Madrid, Madrid Spain; 9 Servicio de Psiquiatría y Psicología Hospital Sant Joan de Déu Barcelona Barcelona, Catalonia Spain; 10 Servicio de Salud Mental Infanto Juvenil ALTHAIA, Xarxa Assistencial Universitaria de Manresa Manresa Spain; 11 Servei de Psiquiatria & Psicologia Infantil Juvenil Hospital Universitari Mútua de Terrassa, Fundació Recerca Mútua de Terrassa Terrassa Spain; 12 Department of Child and Adolescent Psychiatry and Psychology, 2021SGR01319 Institute of Neuroscience Hospital Clínic de Barcelona Barcelona Spain; 13 Neuropsychology and Neuroimaging Group Clinical and Experimental Neurosciences Area IDIBAPS Barcelona Spain; 14 Salut Mental Corporació Sanitària Parc Taulí de Sabadell Sabadell Spain; 15 Department of Clinical and Health Psychology Faculty of Psychology and Speech Therapy Universitat Autònoma de Barcelona Barcelona Spain; 16 Department of Psychiatry Hospital de la Santa Creu i Sant Pau Institut d’Investigació Biomèdica- Sant Pau (IIB-SANT PAU) Barcelona Spain; 17 Data and Signal Processing Group Universitat de Vic - Universitat Central de Catalunya Vic Spain; 18 Department of Psychiatry University of Cambridge Cambridge United Kingdom; 19 Department of Medicine Unit of Psychological Medicine Universitat de Barcelona Barcelona Spain; 20 Institute of Neurosciences Universitat de Barcelona Barcelona, Catalonia Spain

**Keywords:** nonsuicidal self-injury, adolescents and young people, smartphone, randomized controlled trial, RCT, intervention, ecological momentary intervention, app, cognitive behavioral therapy, CBT, dialectical behavior therapy, DBT

## Abstract

**Background:**

Nonsuicidal self-injury (NSSI), defined as the deliberate, self-inflicted damage of body tissue without suicidal intent, is increasingly prevalent among adolescents and young adults and poses a major public health concern. Current treatments are often costly, difficult to access, and not tailored to the specific needs of young people. Mobile health (mHealth) interventions represent a promising avenue for scalable, accessible, and cost-effective support for NSSI, especially when combined with real-time assessments and personalized treatment strategies.

**Objective:**

This randomized controlled trial will evaluate the effectiveness of Kalmer, a brief app-based intervention for reducing NSSI and improving emotional well-being. The study has 2 aims: (1) to evaluate a newly developed app-based intervention for adolescents and young adults engaging in NSSI and (2) to assess predictors of treatment outcomes for this app-based intervention. We hypothesize that participants receiving a mobile app–based brief intervention specifically tailored to address NSSI will show a greater reduction in NSSI frequency at the end of treatment and at follow-up than participants receiving a nonspecific app-based intervention. In this paper, we present our study protocol.

**Methods:**

This 2-arm randomized controlled trial, lasting 6 weeks, will include 240 participants aged 14 to 24 years who engage in NSSI. The intervention app, Kalmer, was developed through iterative consultation with clinical and research experts and guided by survey results and evidence-based frameworks such as dialectical behavior therapy and cognitive behavioral therapy. The intervention will include 5 core components: distress tolerance, emotion regulation, mindfulness and self-compassion, interpersonal regulation, and problem-focused coping. The app will deliver multimedia-based ecological momentary interventions triggered by real-time ecological momentary assessments to tailor support to users’ current contexts and needs. Participants are randomized to receive either the Kalmer intervention or a psychoeducational control app. The primary outcome will be NSSI frequency, assessed through self-report at baseline, 6 weeks after intervention, and at 1-month and 3-month follow-ups.

**Results:**

Ethics approval was obtained in December 2022. As of January 2026, a total of 145 participants had consented to participate in the study and completed baseline assessments. Preliminary data show high app engagement and acceptability and positive user feedback regarding app usability and content. Recruitment is ongoing.

**Conclusions:**

This study will provide evidence on the effectiveness of a mobile app–based intervention for NSSI and will explore potential mechanisms of change, supporting the development of accessible digital mental health tools for adolescents and young adults.

**Trial Registration:**

International Standard Randomized Controlled Trial Number Registry ISRCTN63093907; https://www.isrctn.com/ISRCTN63093907

**International Registered Report Identifier (IRRID):**

DERR1-10.2196/86413

## Introduction

Nonsuicidal self-injury (NSSI) is the deliberate destruction of one’s own body tissue in the absence of suicidal intent and is a growing public health concern, particularly among adolescents and young adults [1,2]. NSSI imposes a substantial burden not only on individuals and their families but also on health care systems and society at large. Prevalence rates suggest that 17.2% of adolescents and 13.4% of young adults in community samples report at least one episode of NSSI, with rates reaching up to 58% in clinical adolescent populations [3,4]. NSSI is associated with potentially negative consequences, including impaired interpersonal, academic, and daily functioning, and is one of the strongest predictors of suicidal behavior [5]. In recognition of its severity, the World Health Organization has identified NSSI as one of the top 5 major health threats to adolescents, and it has been included in the *Diagnostic and Statistical Manual of Mental Disorders, Fifth Edition*, as a condition requiring further research.

NSSI often begins during early adolescence and is frequently associated with chronic psychosocial stress, peer rejection, or family-related neglect [6]. Individuals who engage in NSSI typically report greater psychological distress than their peers, regardless of clinical status [4]. In clinical settings, NSSI is commonly observed in the context of broader internalizing and externalizing conditions such as depression, borderline personality disorder (BPD), and eating disorders [7]. Although NSSI is primarily used to regulate negative emotions, it serves a range of functions, both intrapersonal (eg, to relieve distress) and interpersonal (eg, to communicate needs or influence relationships) [8]. According to the 4-factor model of NSSI, these functions can be either positively or negatively reinforced and often co-occur within the same individual [9].

Despite progress in understanding NSSI, significant gaps remain, particularly in the identification of effective, scalable interventions and the mechanisms underlying treatment response. Established treatments such as dialectical behavior therapy (DBT), cognitive behavioral therapy (CBT), and mentalization-based therapy have shown efficacy in reducing NSSI, especially among young adults with BPD traits, but are resource intensive and limited in accessibility [10-12]. Many adolescents and young adults with NSSI face substantial barriers to accessing care, including stigma, a lack of specialized clinicians, and reluctance to seek face-to-face treatment [13].

In this context, digital mental health interventions, particularly mobile apps, represent a promising avenue for improving access to care. Mobile apps can deliver evidence-based support in a self-guided, scalable, and cost-effective format [14]. Young people report high levels of interest in and engagement with digital tools for mental health. However, most app-based interventions currently available either do not specifically target NSSI or have limited empirical support [15]. Furthermore, very few studies have explored their utility in nonclinical populations, despite the growing prevalence of NSSI in these groups. Importantly, there is also limited knowledge regarding individual-level predictors of treatment response, which hinders the development of personalized digital interventions.

To address these gaps, we have developed Kalmer, a brief mobile app–based intervention designed specifically for adolescents and young adults who engage in NSSI. The app has been developed through a structured process that includes expert consultations and survey-based feedback, ensuring that the intervention is grounded in clinical and empirical knowledge. Kalmer integrates ecological momentary assessments (EMAs) to monitor users’ emotional states in real time and trigger tailored ecological momentary interventions (EMIs) delivered through multimedia formats (eg, video, audio, text, and image-based content).

This paper presents the protocol for a randomized controlled trial (RCT) to evaluate the feasibility, acceptability, and preliminary efficacy of the Kalmer intervention. The study will include both clinical and subclinical participants aged 14 to 24 years with a history of NSSI. The primary aim is to develop Kalmer and assess whether it reduces NSSI frequency. The secondary aim is to identify behavioral predictors of treatment response using self-report and real-time app data. This research has the potential to inform the development of accessible, personalized, and evidence-based digital interventions for young people who engage in self-injury.

## Methods

### Study Design and Intervention Arms

#### Overview

This study is a multicenter, double-blinded RCT evaluating the effectiveness of a brief mobile app–based intervention for NSSI in adolescents and young adults. The study was registered with the International Standard Randomized Controlled Trial Number registry. Participants are being randomized to either an NSSI-specific intervention app (intervention 1) or a nonspecific control app (intervention 2).

#### Intervention 1: NSSI-Specific Intervention App

##### Development and Design of Kalmer

The NSSI-specific intervention app, Kalmer, has been designed as a brief app-based program targeting NSSI in adolescents and young adults. It combines evidence-based components from CBT and DBT, with the aim of expanding users’ repertoire of coping strategies to improve well-being, distress tolerance (DT), autonomy, decision-making, problem-solving, sense of meaning, and effectiveness in social relationships.

**Figure 1:**
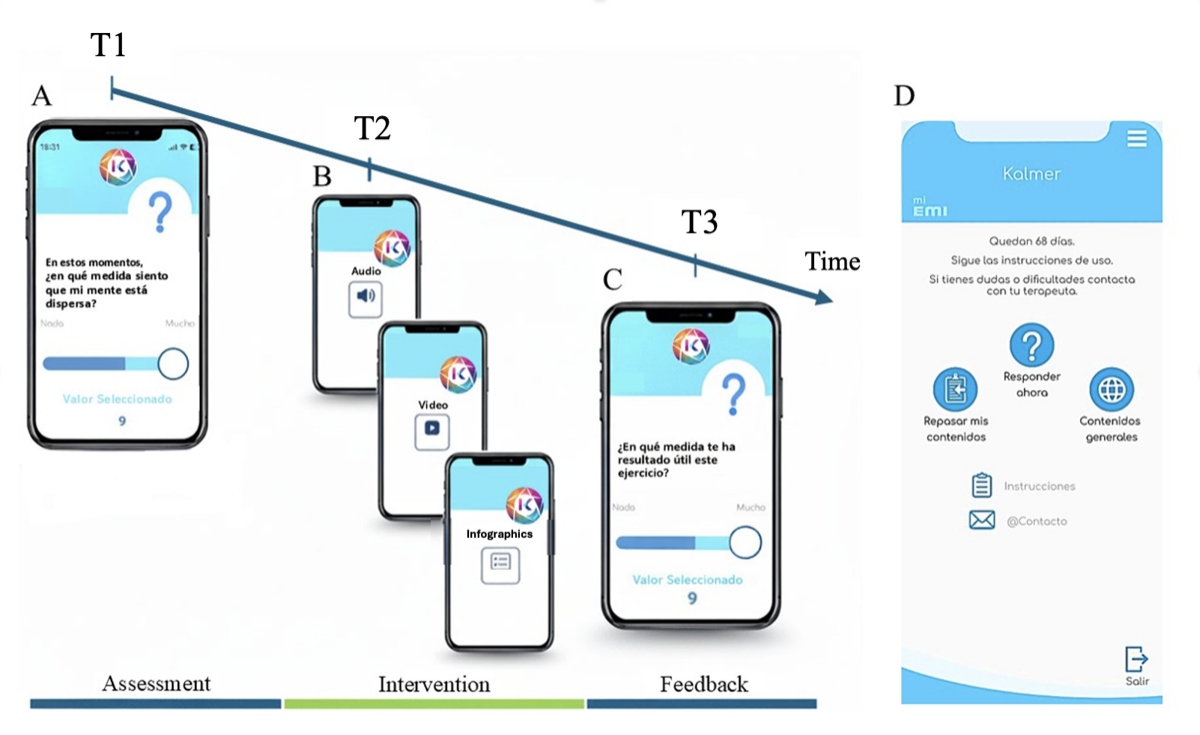
Screenshots of the Kalmer app. (A) Initial ecological momentary assessment (EMA), in this example rating the extent to which the participants feel distracted in the present moment. (B) Based on the EMA response, the app then delivers a tailored ecological momentary intervention (EMI) in a multimedia format, such as audio, video, or an infographic. (C) An EMA is used to collect feedback on the perceived usefulness of the exercise. (D) A screenshot of the main menu of the Kalmer app. T1: time point 1; T2: time point 2; T3: time point 3.

The intervention content was developed through an iterative process involving both clinical and academic experts in NSSI, digital mental health, and adolescent psychopathology. Experts participated in structured meetings and completed targeted surveys to validate and prioritize the core therapeutic elements. This process led to the definition of 5 therapeutic domains that structured the intervention: distress tolerance (DT); mindfulness and self-compassion (M); interpersonal regulation (IR); and problem-focused coping (PC). A schematic representation of the EMA-EMI sequence is shown in Figure 1 (left panel).

##### Ecological Momentary Assessments

EMAs were constructed to gather daily, real-time information on participants’ emotional states, perceived social support, NSSI behaviors, and coping strategies aligned with the 5 therapeutic domains. The assessments were derived from validated psychometric instruments, including the 15-item Five Facet Mindfulness Questionnaire [16,17], the 28-item Coping Orientation to Problems Experienced questionnaire [18,19], the 12-item Interpersonal Support Evaluation List [20,21], the Difficulties in ER Scale (DERS) [22,23], and the Inventory of Statements About Self-Injury (ISAS) [24].

Table 1 shows 2 types of EMAs that were implemented. On-demand EMAs were accessible at any time and provided participants with an immediate, tailored EMI upon completion. Scheduled EMAs were delivered daily at 6:00 PM, with a reminder at 7:00 PM, to ensure consistent engagement. The question sets varied slightly between the on-demand and the scheduled EMAs. Additional items regarding social media use were included in the scheduled EMAs, given their relevance in this age group and their established association with NSSI behaviors [25,26].

**Table 1:** Scheduled and on-demand ecological momentary assessment (EMA) questions.

Component	On-demand EMA	Scheduled EMA
Emotion regulation	At this moment, to what extent do my emotions seem overwhelming to me?	At this moment, to what extent do my emotions seem overwhelming to me?
Interpersonal regulation I	During the day today, to what extent am I spending more time than usual on social media because I feel lonely and/or rejected?	At this moment, to what extent do I feel lonely and/or rejected?
Interpersonal regulation II	N/A^a^	During the day today, how many hours have I spent on social media?
Interpersonal regulation III	N/A	During the day today, to what extent have I spent more time than usual on social media because I have felt lonely and/or rejected?
Mindfulness	At this moment, to what extent do I feel that my mind is wandering?	At this moment, to what extent do I feel that my mind is wandering?
Problem-focused coping	At this moment, to what extent do I feel able to cope with my problems?	At this moment, to what extent do I feel able to cope with my problems?
Distress tolerance	At this moment, to what extent do I think about harming myself?	At this moment, to what extent do I think about harming myself?

^a^N/A: not applicable.

##### Ecological Momentary Interventions

EMAs dynamically trigger EMIs tailored to participants’ real-time needs. EMIs are delivered through multimedia formats, including videos, images, infographics, audio clips, and external links. Each EMI module features a short video with psychoeducation, examples, and simulated testimonies, followed by exercises designed for real-world application.

The first week of app use includes daily introductory videos presenting each therapeutic component and encouraging adherence. Both scheduled and on-demand EMAs and EMIs will be available throughout the intervention period.

The DT domain was prioritized because of its strong association with NSSI behaviors [27]. DT-focused EMIs aimed to help users withstand intense emotional states without resorting to NSSI, emphasizing skills for tolerating, but not necessarily reducing, the intensity of distress until it subsides.

The ER component focused on helping participants understand and modulate strong positive and negative emotions, as emotional cascades are a known trigger for self-injury [27]. The mindfulness and self-compassion domain equipped users with skills to remain present and attuned to their emotions, thereby enhancing the application of other coping strategies.

The interpersonal regulation and problem-focused coping domains address relational difficulties and life challenges that are particularly salient during adolescence and young adulthood [28]. Strategies within these domains aim to mitigate feelings of rejection and abandonment, both of which are linked to NSSI [6,29].

##### Multimedia Content and Prioritization

A total of 32 EMI videos, 50 images, 42 infographics, 8 audio files, and 6 YouTube links were created to explain therapeutic strategies, provide psychoeducation, and encourage skill application. EMIs were prioritized based on expert judgment: when the user reaches the specific scores detailed in Table 2, an EMI will be activated to offer the intervention objects linked with the score and its component. If multiple components warranted intervention, the app delivered the EMI corresponding to the most maladaptive state at that moment. The decision rules for EMI selection and prioritization are detailed in Table 2.

**Table 2:** Ecological momentary intervention (EMI) component order.

EMA^a^ score	EMI component
DT^b^ between 8 and 10	DT content related to doing something else to avoid self-injury, as “STOP^c^,” “opposite action,” and “distraction” techniques, but focused on dealing with NSSI^d^ behaviors
DT between 6 and 7	DT content related to activities or strategies that can be used to delay NSSI behaviors
ER^e^ between 8 and 10	ER content related to doing something else or different, as “STOP,” “opposite action,” and “distraction” techniques
IR^f^ between 8 and 10	IR content related to not acting impulsively, contact with support network and emotion management of difficult situations.
M^g^ between 5 and 10	M content related to practicing mindfulness with different strategies.
ER between 6 and 7	ER content related to reevaluation and relaxation.
IR between 6 and 7	IR content related to how to connect with others
DT between 4 and 5	DT content related to things to do to protect yourself in the event of NSSI behaviors appearing, such as safety plans.
PC^h^ between 0-3	PC content related to the steps to solve problems and to make decisions.
ER between 4 and 5	ER content related to normalizing the emotions felt in the moment.
IR between 4 and 5	IR content related to remind the user to connect with people and that is not alone.
PC between 4 and 6	PC content related to short reminders of how to deal with problems.
SM^i^ between 7 and 10	SM content to explain how social media do not help with rejection feelings.

^a^EMA: ecological momentary assessment.

^b^DT: distress tolerance.

^c^STOP: stop, take a breath, observe, and proceed mindfully.

^d^NSSI: nonsuicidal self-injury.

^e^ER: emotion regulation.

^f^IR: interpersonal regulation.

^g^M: mindfulness.

^h^PC: problem-focused coping.

^i^SM: social media.

The app also featured 2 content libraries. The “Review My Contents” section served as a repository of all previously received EMIs, allowing users to review and practice the strategies at any time. The “General Contents” section provided crisis hotline numbers and links to reputable websites related to self-injury and mental health. Figure 1D shows the 3 principal options displayed in the app: “Answer now,” “Review my content,” and “General content.”

##### Youth Advisory Board

A youth advisory board composed of 9 young adults with lived experience of NSSI provided systematic feedback on the app’s functionality, design, and overall user experience. Their perspectives informed the development of the final version of the Kalmer app.

#### Intervention 2: Nonspecific Control App

Participants assigned to intervention 2 group use the same Kalmer app interface, which adheres to the same EMA schedule as those in intervention 1. However, this version delivers static, nonpersonalized content that is not tailored to individual EMA responses. Specifically, the app comprises 2 psychoeducational modules: (1) an introductory module outlining the app-based intervention and providing general information on NSSI and (2) a module focused on emotional well-being, healthy lifestyle behaviors, and strategies for managing anxiety.

For both intervention 1 and intervention 2, app use metrics (eg, session completion rates and time spent within the app) are being collected continuously. In either condition, any participant reporting recurrent self-injury or heightened clinical risk will prompt the research team to notify the participants’ clinical care team to initiate an appropriate safety protocol. Additionally, both versions of the app include a “General contents” section containing the national suicidal behavior hotline number and links to validated online resources pertaining to self-injury and mental health.

### Participants, Eligibility Criteria, and Recruitment

Recruitment is ongoing, and we aim to recruit a total of 240 participants aged 14 to 24 years. As shown in Table 3, all participants will have engaged in at least 5 episodes of NSSI in the past year and will have access to a smartphone. They must also provide informed consent; parental consent is required for minors. Exclusion criteria for all participants include a current psychiatric crisis or inability to complete study procedures.

**Table 3:** Inclusion and exclusion criteria for the study groups.

Inclusion and exclusion criteria	Study groups	
	Clinical	Subclinical
Aged 14-24 y	✓	✓
Providing informed consent online	✓	✓
Owning a smartphone	✓	✓
Having engaged in NSSI^a^ on ≥5 different days in the last 12 months	✓	✓
Co-occurring with any other diagnosis	✓	
Currently receiving psychological or psychiatric treatment	✓	
Absence of important clinical instability	✓	✓

^a^NSSI: nonsuicidal self-injury.

Participants with NSSI are being recruited from clinical sites (ie, clinical group, n=120) and from the community (ie, subclinical group, n=120). Participants categorized as clinical are receiving treatment from one of the mental health service sites involved in this study, whereas subclinical participants are not receiving treatment from a public mental health unit. Recruitment is being carried out through multiple channels including clinics, schools, social media, and mental health organizations. A dedicated study website provides information, facilitates consent, and guides participants through the screening and assessment process.

### Screening, Baseline, and Follow-Up Assessments

**Figure 2:**
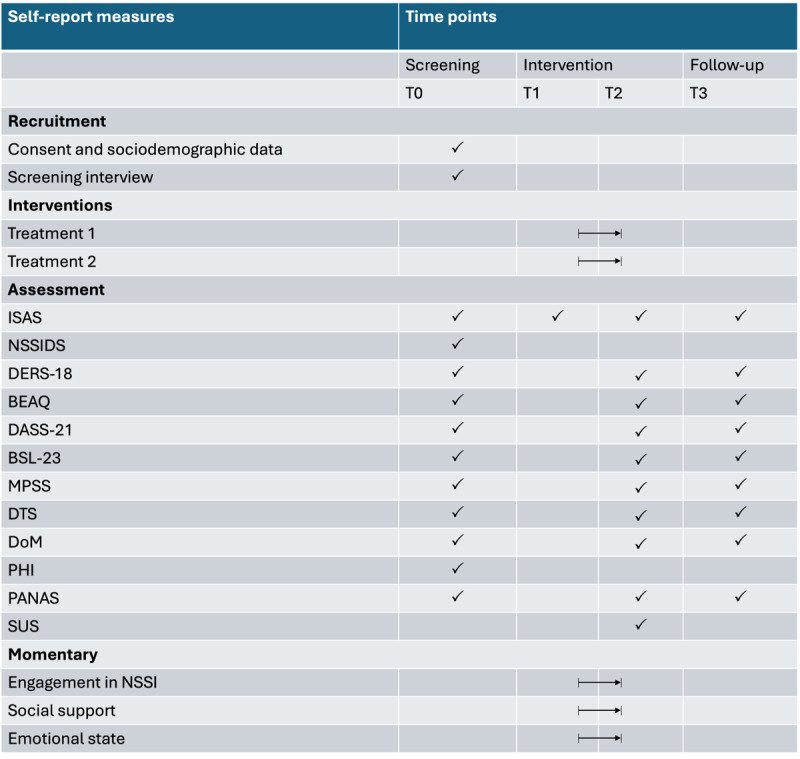
Schedule of self-report measures administered across study time points (T0-T3). BEAQ: Brief Experiential Avoidance Questionnaire; BSL-23: Borderline Symptom List–23; DASS-21: Depression Anxiety Stress Scales; DERS-18: brief version of the Difficulties in Emotion Regulation Scale; DoM: Delusion of Me; DTS: Dialectical Thinking Scale; ISAS: Inventory of Statements About Self-Injury; MPSS: Multidimensional Scale of Perceived Social Support; NSSI: nonsuicidal self-injury; NSSIDS: nonsuicidal self-injury disorder scale; PANAS: Positive and Negative Affect Schedule; PHI: Pemberton Happiness Index; SUS: System Usability Scale.

Interested individuals are being contacted by the study’s research assistant and invited to attend a screening interview, during which they receive detailed information about the study, undergo an eligibility assessment for participation, and provide informed consent in electronic or paper form. For participants younger than 18 years, parental consent is also required. Upon consent, participants complete the online assessment protocol consisting of sociodemographic information and validated self-report questionnaires. Participants are invited to complete online questionnaires at multiple time points throughout the study to assess primary and secondary outcomes and momentary data during the app-based intervention. Figure 2 provides an overview of the instruments administered at each assessment point (T0-T3), including baseline, treatment start, postintervention, and 3-month follow-up evaluations.

### Description of Measures

#### Screening Interview

Questionnaires at the baseline interview assessment include the validated Spanish version of the Patient Health Questionnaire-9 [30-32]. Selected items from the Columbia-Suicide Severity Rating Scale [33] are also used. Finally, the clinician-rated severity of NSSI, a 5-point scale developed to assess the severity of NSSI behaviors experienced by an individual in the past year, is being completed [34].

#### Primary Outcome

The primary outcome is the frequency of NSSI behaviors, assessed using the ISAS [24] at the predefined study time points. At baseline (T0), the ISAS assesses the lifetime frequency and functions of NSSI behaviors [24]. At follow-up assessments, a modified version of ISAS is used to inquire about NSSI behavior in the last month. The Spanish version of the ISAS is used in this study, demonstrating strong internal consistency for both the interpersonal and intrapersonal subscales (Cronbach α=0.87 and 0.90, respectively) [35].

#### Secondary Outcomes

##### NSSI Features

The Nonsuicidal Self-Injury Disorder Scale [36] is a self-report measure designed to assess the proposed criteria for NSSI disorder (as stated in the *Diagnostic and Statistical Manual of Mental Disorders*, *Fifth Edition*) [37]. The Spanish version of the scale shows good internal reliability (Cronbach α=0.88) [36].

##### Emotion Regulation

The brief version of the DERS-18 is the shortened form of the original DERS [22]. The Spanish translation of this scale is being used. The DERS-18 preserves the original 6-subscale structure assessing key domains of ER difficulties, including a lack of emotional awareness, a lack of emotional clarity, nonacceptance of emotions, inability to engage in goal-directed behavior when feeling emotional, engagement in impulsive behavior when feeling emotional, and inability to access ER strategies. The scale demonstrates strong internal consistency, with a total score of Cronbach α=0.91 [38].

The Brief Experiential Avoidance Questionnaire is a 15-item measure assessing an individual’s tendency to avoid or escape from unwanted internal experiences such as uncomfortable emotions, thoughts, memories, or sensations [39]. The Spanish version is being used and shows good internal consistency (Cronbach α=0.82) [39].

##### Symptoms

The Depression Anxiety Stress Scales–21 is a self-report measure that evaluates the frequency and severity of 21 negative emotional symptoms experienced over the past week, organized into 3 subscales: depression, anxiety, and stress. The Spanish version of the scale has shown moderate to good internal consistency, with Cronbach α coefficients of 0.84 for depression, 0.70 for anxiety, and 0.82 for stress [40].

The Borderline Symptom List–23 [41] assesses BPD symptom severity. Participants rate each item on a 5-point Likert scale from 0 (not at all) to 4 (very strong). The Spanish version of the scale is being used, which has high reliability (Cronbach α=0.949) [42].

##### Social Support

Perceived social support is being measured using the Multidimensional Scale of Perceived Social Support [43]. In this study, the validated Spanish version of this scale is being used, which shows good internal reliability (Cronbach α=0.88) [44]. A high score indicates greater social support, and the response format ranges from 1 (completely disagree) to 7 (completely agree).

##### Process Measures

The Dialectical Thinking Scale [45] is a validated 5-item measure assessing dialectical thinking. It consists of a 2-factor structure: “Both Sides,” which reflects the ability to acknowledge and accept opposing viewpoints as equally valid (thereby promoting cognitive flexibility and minimizing polarized thinking) and “Both Sides in Me,” which captures the capacity to incorporate and accept internal contradictions. This scale demonstrates strong psychometric properties, with high internal consistency (Cronbach α=0.81) and sound test-retest reliability (intraclass correlation coefficient=0.82 for “Both Sides” and 0.64 for “Both Sides in Me”).

The Delusion of Me is a 24-item questionnaire assessing a latent construct proposed to unify 3 core mindfulness-related processes: acceptance, decentering, and nonattachment. The general factor showed good reliability, with a hierarchical omega (ωh) of 0.81, indicating that most of the reliable variance in the composite score is attributable to the general construct, rather than to specific subcomponents [46]. Participants rate each item on a 5-point Likert scale ranging from 1 (not at all true or rarely true) to 5 (always or very frequently true).

##### Well-Being

The Pemberton Happiness Index [47] integrates hedonic and eudaimonic aspects of well-being. The Spanish version of the index is being used, which shows high internal consistency (Cronbach α=0.91).

The Positive and Negative Affect Schedule is a 20-item self-report measure assessing positive affect (ie, interested, satisfied, and excited) and negative affect (ie, anxious, guilty, and aggressive). Participants rate each item on a scale ranging from 1 (very slightly or not at all) to 5 (extremely), indicating the extent to which each adjective describes their current mood at the time of completing the questionnaire. The result is the sum of the values. This scale is useful to measure emotional fluctuations throughout a specific period, and a Spanish adaptation is being used [48].

##### User Experience

To evaluate user experience with the app, participants complete the System Usability Scale, a 10-item tool with strong reliability for assessing perceived usability of digital technologies. Items are rated from 1 (strongly disagree) to 5 (strongly agree). The SUS score can be interpreted with a qualitative scale ranging from “worst imaginable” to “best imaginable” usability [49].

##### Daily Assessment

EMAs delivered via the app collect real-time data throughout the intervention phase corresponding to the 5 different EMI domains as previously described in Table 1.

### Randomization and Procedure

**Figure 3:**
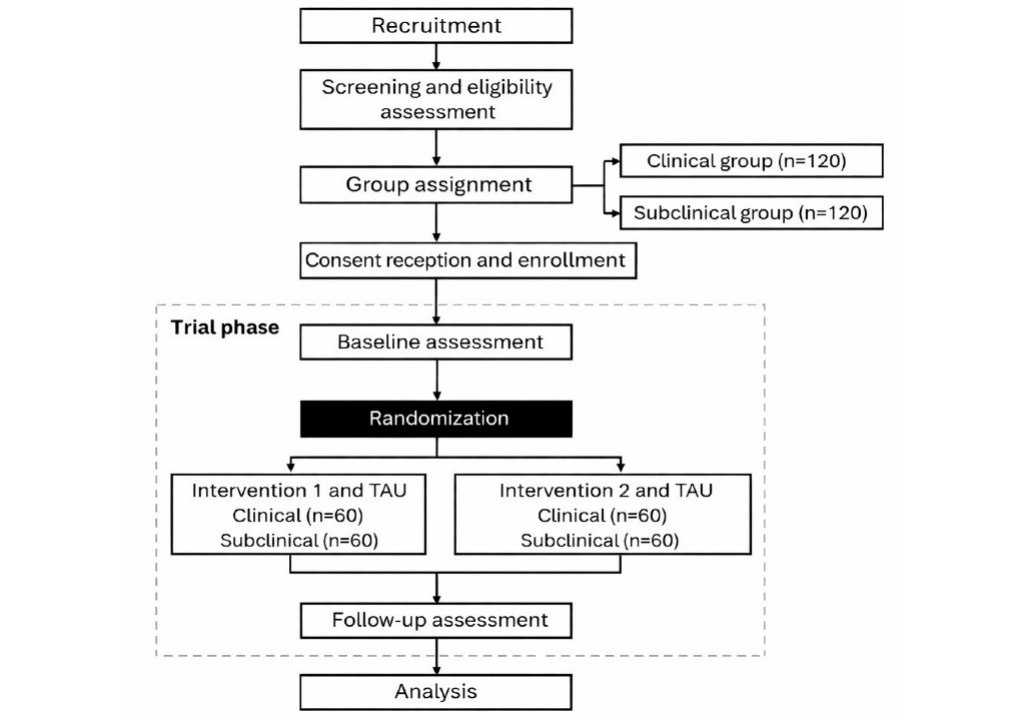
Study flowchart. TAU: treatment as usual.

Figure 3 depicts the evaluation, randomization, and allocation of participants to intervention 1 or intervention 2 between participant groups. Following screening, eligible participants in the NSSI clinical and NSSI subclinical groups are being randomized to either intervention 1 or intervention 2 using an adaptive randomization procedure stratified by age (14-16, 17-19, and 20-24 years) and sex. This approach ensures a balanced distribution across intervention arms. Each treatment group will include 120 participants, divided evenly between the clinical and subclinical groups. In addition, both intervention conditions continue to receive treatment as usual in the NSSI clinical group, which may include psychological or pharmacological care within a public health unit.

Participants receive access to either version of the app, which has been developed for both Android and iOS platforms. To use the app, the user obtains an access code from the researcher, and the app remains active for 6 weeks.

### Sample Size Calculation

Power analyses conducted using G*Power (version 3.1) indicated that, to detect a medium effect size (Cohen *f*=0.27) with 90% power and a significance level of .05, 100 participants per group are required. Allowing for up to 20% attrition, 120 participants will be recruited per intervention arm, resulting in a total NSSI sample of 240 participants.

### Statistical Analysis

Primary analyses will be conducted using linear mixed-effects models to evaluate the impact of the intervention on both momentary and self-report outcomes. Time (T0, T1, T2, and T3) will be included as a within-subject factor, while intervention (intervention 1 vs intervention 2) and group (clinical vs subclinical) will be modeled as between-subject factors. Random intercepts for participants and random slopes for time will be specified. Secondary analyses will explore potential moderators and mediators of treatment response, including app engagement, baseline severity, and demographic variables. Missing data will be addressed using maximum likelihood estimation under the assumption that data are missing at random.

### Ethical Considerations

The study received ethics approval from the Research Ethics Committee of Bellvitge University Hospital on December 12, 2022 (reference PR336/22; CSA PR4/2022). All prospective participants are provided with written information clarifying that their involvement in the study is entirely voluntary. They are informed that choosing not to participate will not result in any negative consequences and that they retain the right to withdraw from the study at any point without penalty. Additionally, participants are given the opportunity to seek clarification or ask any further questions.

Participant privacy and confidentiality are strictly protected. Personal data are pseudonymized and stored on secure, encrypted servers, accessible only to authorized members of the research team. Data handling procedures comply with applicable data protection regulations, including the General Data Protection Regulation (EU 2016/679). No personally identifiable information will be included in study analyses or publications. Participants who complete the full intervention receive financial compensation of €45 (US $52.20). Compensation is provided for time and resources invested and is not contingent on study outcomes.

For ethical reasons, when the group assigned to intervention 2 completes the posttreatment assessment (2 months after the baseline evaluation), the Kalmer app will be offered to participants in the control group if the study shows a significant reduction in the frequency of NSSI.

## Results

This study was funded in 2022 and received ethics approval in December 2022. It was also registered in the UK’s clinical study registry (International Standard Randomized Controlled Trial Number; ISRCTN63093907) in March 2025. As of January 2026, a total of 145 participants have consented to participate in our study and completed baseline assessments. Preliminary data show high app engagement and acceptability and positive user feedback regarding app usability and content. Recruitment is ongoing. We expected to complete recruitment by March 2026 and finish data analysis and publish final results in October 2026. We plan to publish the data and summary findings in a peer-reviewed journal and at conferences.

We anticipate that the results of this study will offer valuable insight into the prevalence and patterns of NSSI in both clinical and nonclinical populations, particularly in the postpandemic context. Using EMAs and self-report measures, we expect to detect real-time fluctuations in emotional states, social experiences, and NSSI urges and behaviors.

The Kalmer app–based intervention, delivered via EMIs tailored to real-time affective states, is expected to reduce the frequency and intensity of NSSI urges and behaviors. Improvements in ER, perceived social support, and self-compassion are also anticipated. Furthermore, we expect the intervention to show high acceptability and usability among young adults with a history of NSSI, as assessed through validated self-report scales.

## Discussion

NSSI is a significant public health concern that typically emerges during adolescence and young adulthood and is associated with a range of negative mental health outcomes [5]. Despite increasing awareness and prevalence, access to evidence-based treatments remains limited [10-12,50]. Traditional psychotherapies such as DBT and CBT have demonstrated efficacy in reducing NSSI but are resource intensive; often inaccessible due to long waitlists, stigma, or a lack of specialized providers; and frequently underused by the populations most affected [51-54].

Digital mental health interventions, particularly mobile app–based formats, offer a promising avenue for addressing these barriers. They provide scalable, cost-effective, and private access to evidence-informed strategies that can be tailored to users’ needs and embedded into daily life [55]. However, few digital tools have been specifically developed or validated for individuals engaging in NSSI, especially in young populations. There is also a lack of evidence regarding their feasibility, acceptability, and the mechanisms through which they might influence self-injurious behavior, although recent studies show promising results, including reductions in NSSI frequency, improvements in ER, and generally positive feasibility and acceptability outcomes [56].

This paper presents the protocol for an RCT to evaluate the feasibility, acceptability, and preliminary efficacy of the NSSI-specific Kalmer intervention. Kalmer is a brief mobile app–based program co-designed with clinical experts and individuals with lived experience of NSSI. It integrates EMAs with real-time EMIs targeting emotional dysregulation through mindfulness-based and self-compassion–based strategies. The app has been designed to engage young users in both clinical and subclinical settings and deliver timely, context-sensitive support.

By assessing usability, app engagement, and changes in NSSI-related thoughts and behaviors, this study aims to generate early evidence on whether such an intervention is viable and beneficial for young people. The inclusion of real-time assessments and personalized content delivery allows for a granular understanding of how emotional states and environmental contexts interact with intervention use and may potentially inform how to optimize timing and modality for future personalization.

Expected results may inform the development of personalized digital care pathways by identifying both stable (eg, personality traits) and dynamic (eg, emotional fluctuations) predictors of intervention response. This approach aligns with contemporary calls for precision mental health frameworks and could help segment users into subgroups most likely to benefit from specific components or delivery modes.

However, some limitations are anticipated. As a pilot trial, the study may be underpowered to detect small-to-moderate clinical effects and may lack a long-term follow-up. Reliance on self-report and EMA data, although informative, introduces potential for missing data and response biases. App engagement may also vary significantly across users, and understanding dropout or nonuse patterns will be critical for informing future iterations. Additionally, although the intervention is informed by established therapeutic models, its brevity and digital delivery format may limit its scope compared with comprehensive face-to-face treatment.

Despite these limitations, this trial represents a meaningful step toward a scalable and accessible intervention for people with NSSI. It leverages real-time data to adapt support to user needs, draws from stakeholder expertise, and targets an underserved but highly affected population. Findings from this feasibility phase will inform iterative improvements to Kalmer and lay the groundwork for future large-scale evaluations of its clinical efficacy and cost-effectiveness.

## Appendix

Multimedia Appendix 1 Peer review report from ‘La Marató de TV3’ Foundation (Catalonia, Spain).

Multimedia Appendix 2 SPIRIT (Standard Protocol Items: Recommendations for Interventional Trials) checklist.

## Abbreviations

BPD: borderline personality disorder
CBT: cognitive behavioral therapy
DBT: dialectical behavior therapy
DERS: Difficulties in Emotion Regulation Scale
DT: distress tolerance
EMA: ecological momentary assessment
EMI: ecological momentary intervention
ISAS: Inventory of Statements About Self-Injury
NSSI: nonsuicidal self-injury
RCT: randomized controlled trial

## Data Availability

The datasets generated or analyzed during this study are available from the corresponding author on reasonable request.
