# Supplementary material for: A Smartphone-Based Psychological Intervention for Nonsuicidal Self-Injury (Kalmer App): Protocol for a Multicenter Double-Blind Randomized Controlled Trial
Source: JMIR Res Protoc. 2026 Jan 27;15:e86413. doi: 10.2196/86413 (PMC12892031; doi:10.2196/86413)
Supplement: Multimedia Appendix 1 [file resprot_v15i1e86413_app1.pdf]

121 – 202233
~~122 – 202212~~

Project ID: **450**
Title: **Design and analysis of the effectiveness of a brief mobile App-based intervention for Non-Suicidal Self-Injury: self-report, momentary and biological predictors of treatment outcomes**

Summary to the applicants:

This excellent proposal describes the design and analysis of the effectiveness of a brief mobile app-based intervention for non-suicidal self-injury (NSSI). In addition, the study aims at identifying:self-report, momentary and biological predictors of treatment outcome.

The proposal addresses an important public mental health topic and the results can be expected to contribute substantially to treat a common but hitherto difficult to manage form of self-injurious behaviour. This type of self-harm (most often cutting or burning themselves) occurs within 13 to 17% of this age group in the general population and up to 58% in clinical samples, as well as being a significant predictor of suicide, and it is therefore a significant public health concern.

For those participants with previous NSSI, the investigators will compare participant wellbeing and quality of life outcomes between those using the NSSI app with those using a non-specific psychoeducation app (matched by age and sex) in order to assess the effectiveness of the NSSI app. The study will include young people who have experienced NSSI from both clinical and non-clinical groups. A healthy control group with no previous NSSI will also be included. These will complete only the functional MRI component.

All participants will have a functional MRI (fMRI) while completing tasks aiming to assess their pain response to noxious pressure and their emotional response to social rejection. The investigators propose that those young people in the NSSI group will report lower responsiveness to pain but higher responsiveness to social rejection, a combination that could assist in maintenance of this physically harmful response to emotional pain. This component of the study aims to contribute to the understanding of the biological mechanisms that underlie NSSI and its treatment, thereby contributing to greater ability to predict NSSI risk based on neuroimaging data or by combining neuroimaging data with other data.

In addition, those using the apps will be asked to complete regular momentary assessments via the app throughout the intervention phase. Machine learning will be used to build predictive models of NSSI to allow the app to provide more individualized interactions with users based on their momentary NSSI risk; these predictive models will also use data gained from the fMRI study.

This is a well-written, well-considered and comprehensive proposal. It attempts to address the issue of treatment of NSSI among adolescents using novel and robust research methods. It seeks to both understand the predictors of NSSI, as well as testing interventions for it. It also attempts to reduce the significant barriers to NSSI intervention faced by young people, including affordability, availability of suitable clinicians, and a reluctance to engage in face-to-face care, particularly for the stigmatized problem of NSSI.

The most notable strengths of this proposal are its multi-method approach, the inclusion of a youth advisory board in development and refinement of the NSSI app; the use of modern techniques to attempt to predict and intervene to reduce NSSI; the inclusion of three groups: clinical group with NSSI, general population group with NSSI, and a no-NSSI control group in the design; use of machine learning using momentary data to attempt to predict immediate risk of NSSI and intervene to prevent it; the well-considered approach to threats to the study,

including likelihood of dropout, and to the duty of care to the participating young people at risk; the diverse expertise of the research team; and the well-considered dissemination plan.

The only potential drawbacks of the study relate to its ambitious intention to recruit 300 participants who may self-harm, the lack of any clear description of evidence-based content for the App and the high cost of the study. There is a concern that the app could be used for commercial purposes after the study is completed given that the study is funded by public moneys.

Nevertheless, results from the current study can be expected to improve the quality of life of people engaging in self-harming behaviours and their satisfaction with psychological treatments. The analysis of predictors of treatment outcomes could help improve the cost-effectiveness of psychological interventions (by guiding clinical decisions) and could help in the development of personalized treatments for these behaviours. New lines of research could emerge from this project. For example, the app-intervention could be applied to other patient groups.

In sum, although the project has some weaknesses, it might be appropriate for funding, since the proposal has multiple strenghts.
